# Complete stranded RNA profiling during early mouse gonad development

**DOI:** 10.1093/narmme/ugaf014

**Published:** 2025-05-02

**Authors:** Fanghong Ou, Zhangting Wang, See-Wing Chan, Kai-Kei Miu, Wai-Yee Chan

**Affiliations:** School of Biomedical Sciences, Faculty of Medicine, The Chinese University of Hong Kong, Hong Kong SAR, China; School of Biomedical Sciences, Faculty of Medicine, The Chinese University of Hong Kong, Hong Kong SAR, China; School of Biomedical Sciences, Faculty of Medicine, The Chinese University of Hong Kong, Hong Kong SAR, China; School of Biomedical Sciences, Faculty of Medicine, The Chinese University of Hong Kong, Hong Kong SAR, China; School of Biomedical Sciences, Faculty of Medicine, The Chinese University of Hong Kong, Hong Kong SAR, China; CUHK-GIBH CAS Joint Research Laboratory on Stem Cell and Regenerative Medicine, The Chinese University of Hong Kong, Hong Kong SAR, China

## Abstract

Sexual dimorphism in mouse gonads becomes evident at around embryonic day (E)12.5, followed by germ cell differentiation. While prior studies have concentrated on protein-coding genes, our research expands this by profiling the complete spectrum of stranded RNAs including long and short RNAs in one preparation. We identified 2419 differentially expressed genes (DEGs) in the comparison between E12.5 and E11.5 mouse gonads, along with 333 and 770 DEGs in E13.5 versus E12.5 and in E14.5 versus E13.5, respectively. A total of 22 RNA types were annotated, highlighting mRNA, tRNA, long non-coding RNA, antisense RNA, small nucleolar RNA, and microRNA as the most significantly varied types. Serial chromosomal ideographs revealed active chromatin hubs encompassing Hox, tRNA, and stefin gene clusters. Chromosomes 11 and 13 exhibited a higher density of DEGs. Notably, some unassigned reads were mapped to the *Sox9* TESCO (testis-specific enhancer core sequence enhancer), with quantitative PCR results confirming elevated expression of TESCO enhancer RNA at E12.5. By integrating data from public databases, we propose potential regulatory networks involving transcription factors, miR6236, Snord33, long intergenic non-coding RNA *Neat1*, *Anks1b*, and *Lars2*. Our study provides the first complete stranded RNA profiling during early gonad development and serves as a reference for future functional genetic and epigenetic research in reproductive biology.

## Graphical Abstract

**Graphical Abstract:**
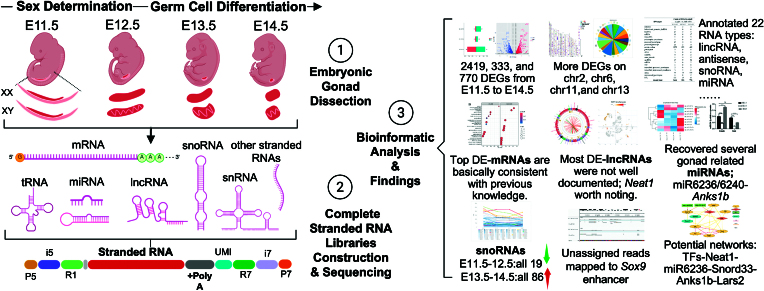

## Introduction

Early bipotent gonads or genital ridges, which are the origin of testis or ovaries, are paired structures raised from proliferating coelomic epithelium at ∼4 weeks post-coitum in humans and embryonic day (E)9.5 in mice [1]. Sexual dimorphism in the XX and XY gonads becomes evident at around E12.5 in mice and at 6 weeks of gestation in humans, followed by initiation of germ cell development [1–4].

Previous studies have demonstrated the significance of various transcription factor (TF) genes, such as *WT1*, *SRY*, *NR5A1*, *SOX9*, and *DAX1*, in developing gonads [5–9]. In recent years, evidence suggests that non-coding RNAs also play crucial roles in gonad differentiation. For example, *Start* (Steroidogenesis activating lncRNA in testis) regulates steroidogenesis specifically in mouse Leydig cells [10]. MicroRNA-202 maintains spermatogonial stem cells and is downstream of *SOX9* in Sertoli cells during testis differentiation [11]. However, we are still far from having a complete picture of the diversity and functional networks of different RNA biotypes in mammalian early gonad development.

The classification of RNA encompasses various types of RNA molecules, each with distinct functions and characteristics. The widely studied RNA types include mRNA, tRNA, rRNA, microRNA (miRNA), small nuclear RNA (snRNA), small nucleolar RNA (snoRNA), small interfering RNA (siRNA), circular RNA (circRNA), and long non-coding RNA (lncRNA) [12, 13]. Long intergenic non-coding RNA (lincRNA) and antisense transcripts are the major functional subtypes of lncRNA.

lncRNAs are generally involved in transcription regulation together with miRNAs. They act as competing endogenous RNAs (ceRNAs) or natural miRNA sponges to balance the influence of miRNAs [14]. miRNAs are transcribed by RNA polymerases II and III as long primary precursor miRNAs (pri-miRNAs) and undergo a series of cleavage events by the ribonucleases Drosha and Dicer to form mature miRNAs containing 21–23 nucleotides. The mature miRNA can be incorporated into the RNA-induced gene silencing complex (RISC) and guides the complex to the target mRNA via complementary base pairing, leading to gene silencing by cleavage, destabilization, or inhibition of translation [15].

snoRNAs are typically 60–300 nucleotides in length. Small Cajal body-specific RNA (scaRNA) is a subtype of snoRNA concentrated at Cajal bodies and follows the classification by box C/D and box H/ACA [16]. Their primary function is to guide chemical modifications of other RNAs by forming functional small nucleolar ribonucleoproteins (snoRNPs). Box C/D snoRNAs guide 2′-*O*-ribose methylation, while box H/ACA snoRNAs guide pseudouridylation [17]. Emerging studies have also implied that snoRNAs might play miRNA-like roles in gene silencing and alternative splicing regulation [18].

In this study, we conducted SEQuoia complete stranded RNA-seq using pooled total RNAs from mouse male and female gonads at E11.5, E12.5, E13.5, and E14.5. By adding poly(A) tails and unique molecular identifiers (UMIs) to all stranded RNAs, we were able to quantify short- and long-stranded RNAs with or without poly(A) tails in one preparation for each time point. This is the first profiling of complete stranded RNAs in early mouse gonad development including sex determination and germ cell development initiation stages.

## Materials and methods

### Animals

Animal experiments followed protocols approved by the Animal Experiment Ethics Committee (AEEC 19/203/MIS and 21-299-MIS) of The Chinese University of Hong Kong (CUHK) and the Guidelines of Animals (Control of Experiments) Ordinance (Cap. 340) licensed by the Department of Health of HK SAR. Mice were housed at CUHK Laboratory Animal Services Centre with a 12 h light and dark cycle, temperature range of 22–24°C, and *ad libitum* access to food.

### Sample preparation

Embryos were collected from pregnant C57BL/6J wild-type mice at E11.5, E12.5, E13.5, and E14.5. For the embryos at E11.5, whole genital ridges (including gonad and mesonephros) were collected. Gonads were dissected free of the mesonephros starting from E12.5 [19]. The samples were stored at –80°C for subsequent steps. For next-generation sequencing (NGS) library construction, at least six male and six female gonads from two litters at the same time points were pooled together for total RNA extraction with Trizol reagent (Invitrogen). For quantitative real-time PCR, male and female gonadal RNAs were extracted separately. All of the total RNA samples were evaluated with Nanodrop 2000 (Thermo) with an *A*_260_/*A*_280_ ratio of ∼2. The RNA integrity number (RIN) values of the samples tested by Agilent 4200 RNA ScreenTape ranged from 8.4 to 10.

### Library construction and sequencing

Libraries were prepared with the SEQuoia Complete Stranded RNA Library Prep Kit (BioRad #17 005 726), SEQuoia Dual Indexed Primers Set (BioRad #12 011 928), and rRNA Depletion Kit (NEB #E6350) with RNA Sample Purification Beads (NEB #E6350S) according to the manufacturer's manual. For each time point, a single library was prepared from a 1 μg total RNA sample from the pooled gonads. The four total RNA samples were first hybridized with the rRNA-targeted probes, followed by RNase H digestion, DNase I digestion, and RNA purification using beads. Ribodepleted RNA samples then underwent fragmentation, end repair, poly(A) tailing, and continuous synthesis steps for cDNA library preparation. The cDNA samples were further purified, concentrated, amplified, and cleaned up. Sequencing was finished by Medikonia Limited using the Hiseq-PE150 platform. Each library was pulled for 15G of raw data.

### Bioinformatic analysis

The BioRad SeqSense Analysis Toolkit (https://github.com/BioRadOpenSource/SEQuoia-Complete) that incorporates fastQC, debarcode, cutAdapt [20], starAlign [21], umiTagging, deduplication with UMI-tools [22], picardAlign, splitBamLong and splitBamMi with Bedtools [23], countLongRNA and countMicroRNA with featureCount [24], and calcRPMKTPM in a container was used for secondary analysis according to the designers’ manual. The reference genome was the mm10 version. Differential expression analysis was conducted with the edgeR package [25] using count matrices as input and filtration threshold |logFC (fold change)| ≥ 1 and FDR (false discovery rate) ≤ 0.05. Chromosome ideograms were plotted with the RIdeogram package [26]. Gene Ontology (GO) and Kyoto Encyclopedia of Genes and Genomes (KEGG) enrichment and gene set enrichment analysis (GSEA) were conducted with clusterProfiler in R studio [27]. Heatmaps were generated with the BGI Dr. Tom platform (https://eu-biosys.bgi.com/#/report/login) with transcripts per million (TPMs) standardized by *Z*-score by row. ceRNA relationships were also analyzed with the Dr. Tom platform. Protein–protein interaction (PPI) prediction was based on STRING11 [28]. TargetScan [29], Starbase [30], RNAhybrid [31], and miRanda [32] were used to predict the target relationship of miRNA. Only the target relationships supported by at least two software systems were presented. The selective networks were visualized with Cytoscape software [33]. Single-cell-related analyses were conducted based on the TEDD Website [34].

### Quantitative real-time PCR

For miRNAs, primers were designed using miRprimer3. Reverse transcription was performed with *Escherichia coli* poly(A) polymerase and the M-MuLV reverse transcriptase-based system (NEB). For other RNAs, primers were designed using NCBI primer blast, or from Primer Bank and Origene. Reverse transcription was performed using the RT Master Mix kit (Takara) following the standard manual. Quantitative PCR was performed using Universal SYBR Green Master mix (Applied Biosystems) and the ABI QuantStudio 7 (Flex) real-time PCR system (Applied Biosystems). All samples were performed in triplicate. Gene expression was normalized to Gapdh or U6 snRNA. Statistical analyses, including Student's *t*-test and two-way analysis of variance (ANOVA), were performed using GraphPad Prism, with *P* < 0.05 considered statistically significant. Primers are listed in Supplementary Table S5.

## Results

### Stranded RNA profiles during early gonad development

**Figure 1.**
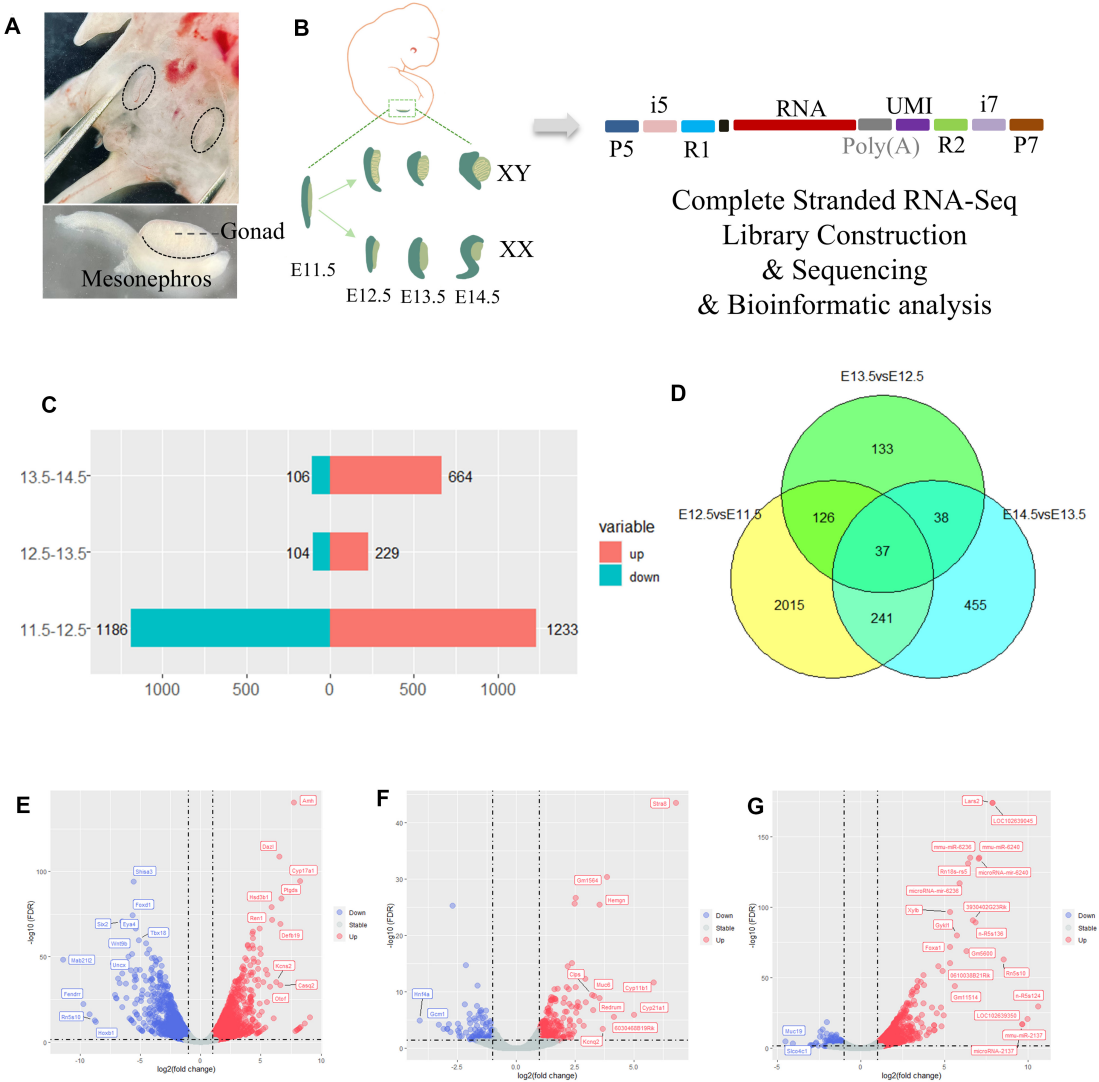
Identification of differentially expressed stranded RNA. (**A**) Illustration of gonads in an embryo. (**B**) Schematic diagram of conducting complete stranded RNA-seq of mouse gonads. (**C**) The numbers of up- and down-regulated differentially expressed genes (DEGs) from E12.5 versus E11.5, E13.5 versus E12.5, and E14.5 versus E13.5. DEGs were analyzed with edgeR, filtrated with |logFC| ≥ 1, FDR ≤ 0.05. (**D**) Venn plot of DEGs across the time points using edgeR. ( **E–** **G**) Volcano plots of DEGs. The *x*-axis represents the FC of the difference after conversion to log2, and the *y*-axis represents the significance value after conversion to –log10. Red represents DEG up-regulated, blue represents DEG down-regulated, and gray represents non-DEGs.

Aiming to profile comprehensive stranded RNA biotypes during early gonad development, we dissected embryonic gonads from wild-type C57BL/6J mice at E11.5–E14.5 for complete stranded RNA-seq library construction, followed by NGS (Fig. 1A, B). The sequencing raw data were processed according to the Bio-Rad Seqsense Analysis Toolkit and yielded 57 million to 76 million unique mapped reads (∼80% of the total) at each time point. A total of 30 462 long RNAs and 635 miRNAs were identified from about half of the unique mapped reads.

Differentially expressed gene (DEG) analysis using the edgeR package revealed that the gonadal transcriptome changed more dramatically during the sex determination stage from E11.5 to E12.5, with 1233 genes up-regulated and 1186 genes down-regulated when applying the filtration threshold as |logFC| ≥ 1 and FDR ≤ 0.05. In contrast, the profiles remained stable from E12.5 to E13.5, with only 330 genes up-regulated and 164 genes down-regulated. Then in the germ cell development stage, from E13.4 to E14.5, the gonadal transcriptome showcase a new profile with 664 genes up-regulated and 106 genes down-regulated (Fig. 1C; Supplementary Tables S1–S3). The Venn plot showed that only 37 genes retained a dynamic change during E11.5–E14.5, while 2015 genes specifically changed from E11.5 to E12.5, and 455 genes specifically changed from E13.5 to E14.5 (Fig. 1D). The difference between developmental stages is even more pronounced from DEGs filtrated with |logFC| ≥ 2 and FDR ≤ 0.05. These findings suggested that the genetic profiles varied greatly between the stages of sex determination and germ cell development.

The DEGs were categorized into 22 RNA biotypes. Protein-coding mRNAs made up the majority of the DEGs. Other significant RNA types included tRNA, lincRNA, antisense RNA, snoRNA, and miRNA (Table 1). Additionally, > 100 DEGs were uncharacterized and labeled as N/A, suggesting that there is still much to be discovered and understood about the RNA regulatory network in gonadal development.

**Table 1.** Classification of differentially expressed stranded RNAs

RNA types	Count of DEGs by edgeR					
	(|LogFC| ≥ 1, FDR ≤ 0.05)			(|LogFC| ≥ 2, FDR ≤ 0.05)		
	E11.5–12.5	E12.5–13.5	E13.5–14.5	E11.5–12.5	E12.5–13.5	E13.5–14.5
Antisense	22	2	4	13	0	2
bidirectional_promoter_lncRNA	1	0	0	1	0	0
lncRNA	27	3	5	14	1	2
miRNA	11	0	17	5	0	10
misc_RNA	1	0	3	0	0	1
polymorphic_pseudogene	1	0	0	1	0	0
processed_pseudogene	25	1	8	8	0	3
processed_transcript	21	2	4	7	0	2
protein_coding	2053	293	402	813	57	133
Pseudogene	2	0	0	0	0	0
rRNA	3	0	6	3	0	4
scaRNA	0	0	2	0	0	0
sense_intronic	1	1	2	0	0	1
snoRNA	19	0	84	0	0	29
snRNA	3	0	7	0	0	3
TEC	1	0	0	0	0	0
transcribed_processed_pseudogene	2	0	3	0	0	0
transcribed_unprocessed_pseudogene	3	2	0	3	1	0
tRNA	46	10	158	3	0	28
unprocessed_pseudogene	6	0	2	2	0	0
N/A	171	19	63	74	6	23
**Grand total**	**2419**	**333**	**770**	**947**	**65**	**241**

### Chromosomal plots revealed active chromatin hubs

**Figure 2.**
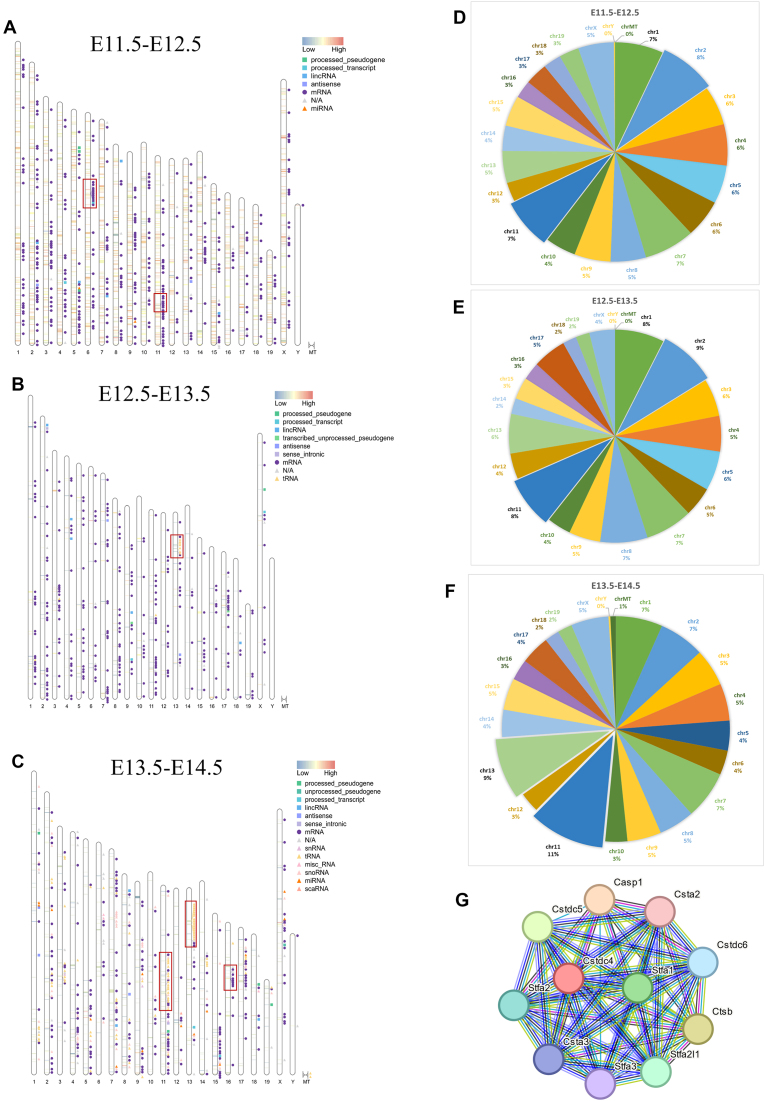
Chromosomal distribution of DEGs. (**A–** **C**) Ideograms of the distribution of DEGs on each chromosome for E12.5 versus E11.5, E13.5 versus E12.5, and E14.5 versus E13.5, respectively. The ideograms were generated with the RIdeogram package in RStudio. Background with colors indicates regions of DEGs with |logFC| ≥ 1 and FDR ≤ 0.05. The darker the color, the greater the difference in up-regulation or down-regulation. Only biotypes of DEGs with |logFC| ≥ 2 and logCPM ≥ 2, FDR ≤ 0.05 were labeled for (A), and only biotypes of DEGs with |logFC| ≥ 1.5 and FDR ≤ 0.05 were labeled in (C). All 333 DEGs from E12.5 to E13.5 were labeled in (B). (**D–** **F**) Pie charts showing the portions of DEGs on each chromosome. (**G**) Networks between stefin genes from the String database.

To better discover the dynamics during early gonad development, the distribution of the DEGs was plotted on the mouse karyotype using the RIdeogram package in RStudio (Fig. 2A–C). Background with colors indicates chromosomal regions of the DEGs. To avoid overly dense coverage of labels, only ∼400 DEGs were labeled with RNA types in each diagram. The numbers of DEGs distributed on each chromosome were summarized in Supplementary Table S4. Chromosomes 2, 7, 11, and 13 exhibited a higher density of DEGs. The proportion of DEGs in most chromosomes showed little variation from E11.5 to E14.5. However, the proportion of DEGs on Chr11 increased from 7.4% to 10.78%, while those on Chr13 rose from 4.71% to 8.70%. In contrast, the proportion of DEGs on Chr6 decreased from 5.58% to 3.51%, and on Chr2 declined from 8.23% to 6.49% (Fig. 2D–F). These results suggested that Chr6 and Chr2 might be more essential in early gonadogenesis and sex determination, while Chr11 and Chr13 might be the most active chromosomes during mouse early gonad development, especially in germ cell development.

We noticed some condensed hubs on Chr6 and Chr11 containing multiple DEGs from E12.5 versus E11.5 (Fig. 2A), including *Hoxa2*–*Hoxa11* except for *Hoxa8*, and processed transcript *Hoxa11os*, which was transcribed from the opposite strand of *Hoxa11*. *Hoxa11* and *Hoxa11os* were the most significant down-regulated *Hoxa* genes from E11.5 to E12.5. *Hoxb2*–*Hoxb8 and Hoxb5os* consisted of another condensed hub across the genome. Down-regulation of the *HoxA* and *HoxB* genes demonstrated the shift from early organogenesis to late developmental programs [35].

During E12.5 to E13.5, an interesting hub appeared on Chr13 containing seven differentially expressed (DE) tRNA genes (Fig. 2B). Several other tRNA hubs on Chr11 and Chr13 emerged during E13.5 to E14.5. The 158 DE tRNAs comprised 20% of the total DEGs during E13.5 to E14.5 and showed 2- to 4-fold up-regulation. Conversely, only 46 tRNAs (2% of total DEGs) showed significant differential expression from E11.5 to E12.5, and they were all down-regulated. These findings demonstrated that germ cell development relies on the early accumulation of tRNAs for a new wave of transcriptome dynamics. The 158 tRNAs can be categorized into 16 groups according to the amino acids they carry. Twenty-three DE tRNAs were responsible for arginine, making up the largest portion of the DE tRNAs (Table 2). n-Te15, n-Te18, n-Te21, and Trnae-uuc that transport lysine were the most abundant tRNAs. These results implied that lysine and arginine might play more essential roles in germ cell development initiation.

**Table 2.** Classification of the amino acids corresponding to differentially expressed tRNAs

Amino acid	E11.5–E12.5	E12.5–E13.5	E13.5–E14.5
Alanine	2	0	12
Arginine	6	0	23
Asparagine	0	0	2
Aspartate	0	0	2
Cysteine	1	0	6
Glutamate	5	1	11
Glutamine	5	1	9
Glycine	0	0	9
Histidine	8	0	13
Isoleucine	3	0	3
Leucine	5	1	8
Lysine	1	1	6
Phenylalanine	2	0	5
Serine	4	1	17
Threonine	0	2	14
Valine	4	3	18
**Grand total**	**46**	**10**	**158**

Another dense hub during E13.5 to E14.5 was found on Chr16, with one highly abundant ncRNA LOC102639045 (Gm15564), and six protein-coding genes, *Stfa2l1*, *Cstdc4*, *Stfa1*, *Cstdc6*, *Cstdc5*, and *Stfa3*,expressed at a low level (Fig. 2C). The uncharacterized LOC102639045 showed dramatic down-regulation from E11.5 (TPM 5963.93) to E13.5 (TPM 350.78) and then up-regulation to the highest expression at E14.5 (TPM 26 095.28) in our datasets. The six adjacent coding genes showed strong connections as parts of the stefin gene cluster (Fig. 2G), which encode cysteine protease inhibitors that play important roles in normal cellular functions such as protein turnover, antigen processing, and apoptosis [36, 37]. There might be potential regulatory networks between LOC102639045 and the stefin genes during gonad development.

These chromosomal level results provide a valuable reference map for future study on developmental and reproductive biology and can advance prenatal screening and diagnosis, and genetic counseling, which currently rarely incorporate non-coding regions [38, 39].

### Characterization of DE mRNAs

Gonadal transcriptomic profiling using different methods, such as microarrays [40], serial analysis of gene expression (SAGE) [41], bulk RNA-seq [42], and single-cell RNA-seq [5], has provided extensive mRNA data. Our DEG analysis also recovered numerous coding genes that are known to play important roles in gonad development, such as *Amh* (anti-Mullerian hormone), which prevents Mullerian ducts from developing into the female reproductive tract in XY embryos [43]; *Cyp17a1* (steroid 17-alpha hydroxylase), which is essential in steroidogenesis [44]; and *Dazl*, which belongs to the *Daz* (Deleted in AZoospermia) gene family that encodes an RNA-binding protein in germ cells of males and females (Fig. 1E–G) [45]. The significant up-regulation of these genes supports their importance in early gonad development [46].

*Mab21l1*, *Six2*, and *Wnt9b* were the top three significantly down-regulated genes from E11.5 to E12.5. *Mab21l1* was expressed at a low level at E11.5 and then almost disappeared at the later time points. Unique expression of *Mab21l1* in the lens vesicles and genital tubercle has been reported [47], indicating that it may contribute specifically to early gonad formation and sex determination but not later germ cell and somatic cell maturation. *Six2*and *Wnt9b* have been shown to be related to balancing progenitor cell expansion and differentiation during kidney development [48]. They might contribute to the differentiation and maturation of gonads by a similar mechanism.

**Figure 3.**
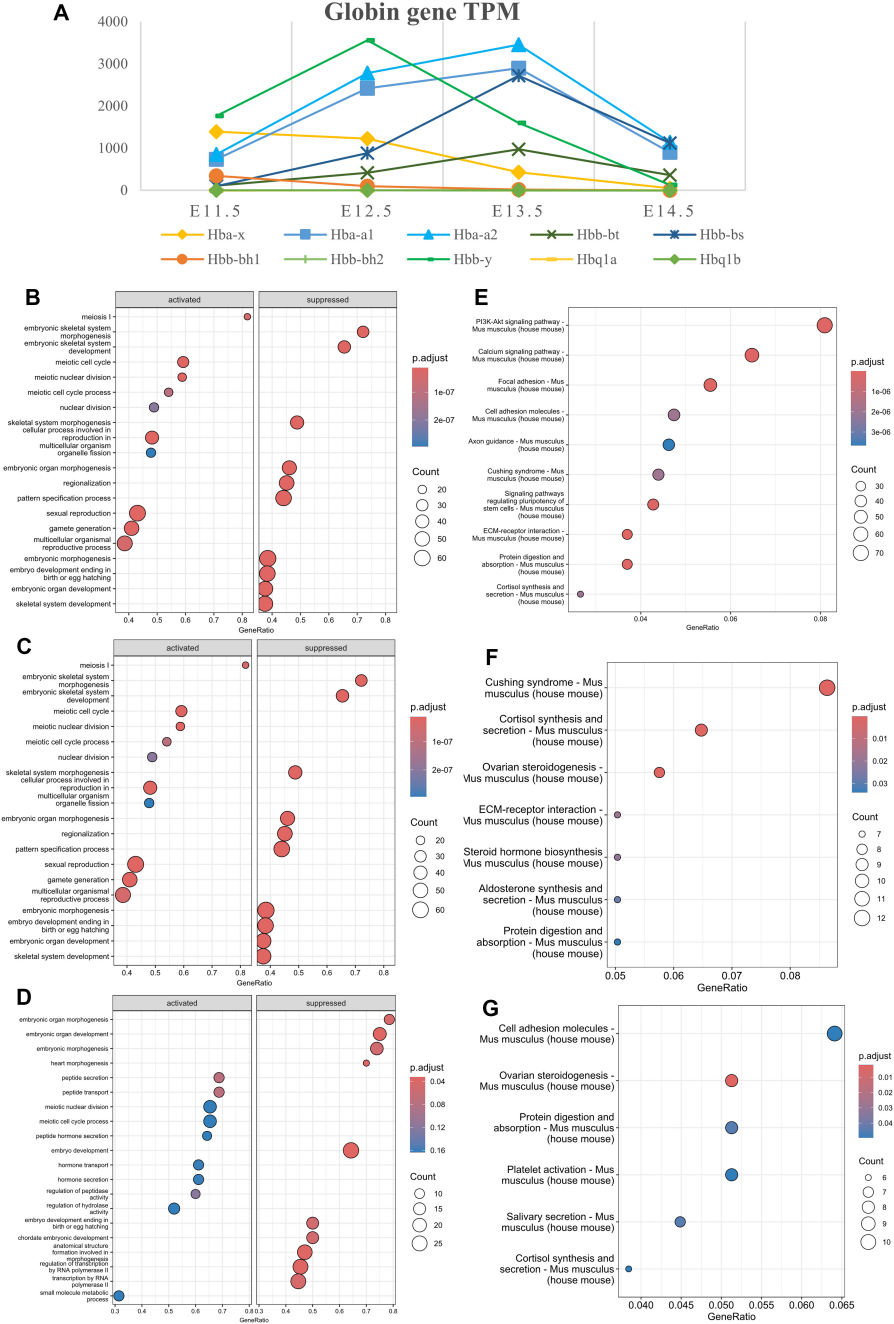
Characterization of DE mRNAs. (**A**) Expression patterns of globin genes during E11.5 to E14.5. (**B–** **D**) GSEA using GO for DEGs of E12.5 versus E11.5, E13.5 versus E12.5, and E14.5 versus E13.5. (**E–** **G**) KEGG enrichment analysis of DEGs of E12.5 versus E11.5, E13.5 versus E12.5, and E14.5 versus E13.5.

GSEA using GO showed that the down-regulated genes were enriched in skeletal development, embryonic organ morphogenesis, regionalization, and pattern specification, which is consistent with the concept that primordial germ cell (PGC) migration and gonad morphogenesis were almost completed at around E11.5. The up-regulated DEGs were mainly associated with meiosis and the reproduction process, indicating that meiosis-related genetic networks may have already been activated at E12.5 (Fig. 3B). KEGG enrichment analysis of the DEGs revealed that these genes play a significant role in regulating sexual dimorphism, mainly through PI3K–Akt signaling and calcium signaling pathways (Fig. 3E).

During E12.5 to E13.5, the top up-regulated genes were *Stra8* (stimulated by retinoic acid gene 8), *Gm1564* (Meioc, meiosis-specific with the coiled-coil domain), *Cyp11b1* (11-beta-hydroxylase), and *Cyp21a1* (21-hydroxylase)*. Stra8* and *Meioc* are both involved in meiosis in germ cell development. *Cyp11b1* and *Cyp21a1* are involved in the production of steroid hormones, especially testosterone and estrogen. The top down-regulated genes were mainly those encoding TFs, including *Hnf1b* (HNF1 homeobox B), *Gcm1* (glial cells missing homolog 1), and *Hnf4a* (hepatic nuclear factor 4, alpha) (Fig. 1D). When sorting the list by logCPM (counts per million), the embryonic globin genes *Hba-x* and *Hbb-bh1* were the top two down-regulated genes with higher expression levels. The globin genes encode different subunits of hemoglobin that are responsible for oxygen transport in red blood cells. Our data showed a transition of the main globin in gonads from *Hba-x*-encoded zeta globin, and *Hbb-y*- and *Hbb-bh1*-encoded epsilon globin to *Hba-*encoded alpha globin and *Hbb*-encoded beta-globin in gonads from E12.5 to E13.5 (Fig. 3A), which was consistent with previous knowledge of globin switches based on liver, heart, and bone marrow research [49, 50].

In the later E13.5–E14.5, most DEGs showed up-regulation rather than down-regulation (Fig. 1G). *Lars2*, which encodes mitochondrial leucyl-tRNA synthetase, exhibited the most dramatic increase at E14.5. Shujun *et al.* reported that *LARS2* regulates apoptosis in ovarian granulosa cells through reactive oxygen species (ROS)-mediated mitochondrial dysfunction and endoplasmic reticulum (ER) stress in patients with premature ovarian insufficiency (POI) [51]. Another significantly up-regulated gene was *Gykl1*, which encodes a glycerol kinase-like protein and is also located in the mitochondrion. *Gykl1* or *Gk2* cooperate with Pld6 to regulate sperm mitochondrial sheath formation and male fertility [52]. Mitochondrial disorders have been linked to several reproductive dysfunctions, such as hypospermatogenesis and primary ovarian insufficiency [53].

Sex chromosome-linked genes are indispensable in regulating sexual development. *Nxf2* (nuclear RNA export factor 2), *AV320801* (PRAME like 3E), *Ctag2l1* (CTAG2 like 1), and *Dcaf8l* (DDB1 and CUL4 associated factor 8 like) that are located on the X chromosome and Usp9y (ubiquitin-specific peptidase 9) that is located on the Y chromosome continued to be up-regulated from E11.5 to E14.5. They also showed biased expression in adult testis [54], implying their male development-specific roles. *Rhox5*, *Rhox6*, and *Rhox9*, which are members of the *Rhox* reproductive homeobox gene family located on the X chromosome [55], increased from E11.5 to E12.5 and then decreased from E13.5 to E14.5, suggesting that the *Rhox* genes may actively function transiently during E11.5 to E13.5 for sex determination and early male and female germ cell development initiation.

GO enrichment analysis demonstrated that the DEGs mainly participated in the meiotic and extracellular matrix (ECM) after E12.5 (Fig. 3C, D). Enrichment of activated genes in peptide secretion and transportation further implied active intercellular communication and remodeling of the ECM after sex determination (Fig. 3D). KEGG enrichment also implied that these genes were mainly involved in ovarian steroidogenesis, cortisol synthesis and secretion, and protein digestion and absorption, confirming the active hormonal signaling during gonadal cell lineage progression (Fig. 3F, G).

Our results of protein-coding mRNAs recovered numerous important genes that contribute to early gonad development, supporting the reliability of our analysis. Additionally, we identified potential key regulators such as *Mab21l1* and *Lars2*, whose functions in early gonad development have not been extensively characterized.

### Characterization of DE lncRNAs

The lncRNA candidates that displayed the greatest up-regulation in sex determination included LOC102635594 (Gm32885), a predicted ncRNA gene that showed biased expression specifically in the adult placenta and testis in RNA profiling datasets from the Mouse ENCODE project [54], suggesting that the lncRNA Gm32885 may specifically function in maternal and embryonic signaling transduction and testis differentiation. Gm15529, which is a HORMA domain-containing 1 pseudogene, was also significantly up-regulated during sex determination. It was predominantly detected in mouse gonads from and after E11.5 in the Mouse Genome Database (MGD) (E-MTAB-6798) [56]. The HORMA domain acts as an adaptor that recruits other proteins and is involved in various cellular processes, including mitotic checkpoints, chromosome synapsis, and the spindle assembly checkpoint [57, 58]. However, the connection between the pseudogene and the related protein-coding genes remains poorly understood.

*Fendrr* and LOC102637974 (Gm34654) were the most significant down-regulated lncRNAs from E11.5 to E12.5. *Fendrr* is believed to function through binding to the histone-modifying complexes PRC2 and/or TrxG/MLL to encourage methylation of target gene promoters, leading to decreased expression of these genes [59]. Thus, a decrease of lncRNA *Fendrr* after E11.5 might be essential for promoting chromatin openness and supporting the new transcriptome profile activation. For genes on sex chromosomes, we found LOC102639708 (Gm21758), the only DE ncRNA located on the Y chromosome during E11.5–E12.5. It was detected specifically in adult testis in the Mouse ENCODE project [54]. On the X chromosome, more uncharacterized lncRNAs were annotated, including up-regulated *Mageb10-ps*, LOC102633678, *Kis2*, *Itih5l-ps*, *Gm715*, *Gm14812*, *Gm6023*, *AA414768*, and *Mdrl* (2310010G23Rik, mitochondrial dynamic-related lncRNA), and down-regulated 5330434G04Rik, LOC102636644, and LOC10105594. Most of the non-coding genes were not widely documented, implying the complexity of non-coding gene networks during sex determination.

While many DE lncRNAs during E11.5–E12.5 were predominantly or specifically detected in the testis [54], the DE lncRNAs during E12.3–E13.5 exhibited ubiquitous expression in different tissues, such as the up-regulated antisense 4833422C13Rik and lincRNA *Gm6297* and down-regulated *Lncenc1* and *Gm15564*. These DE ncRNAs are likely to contribute to fundamental biological processes during this relative quiescent stage of early gonad development.

**Figure 4.**
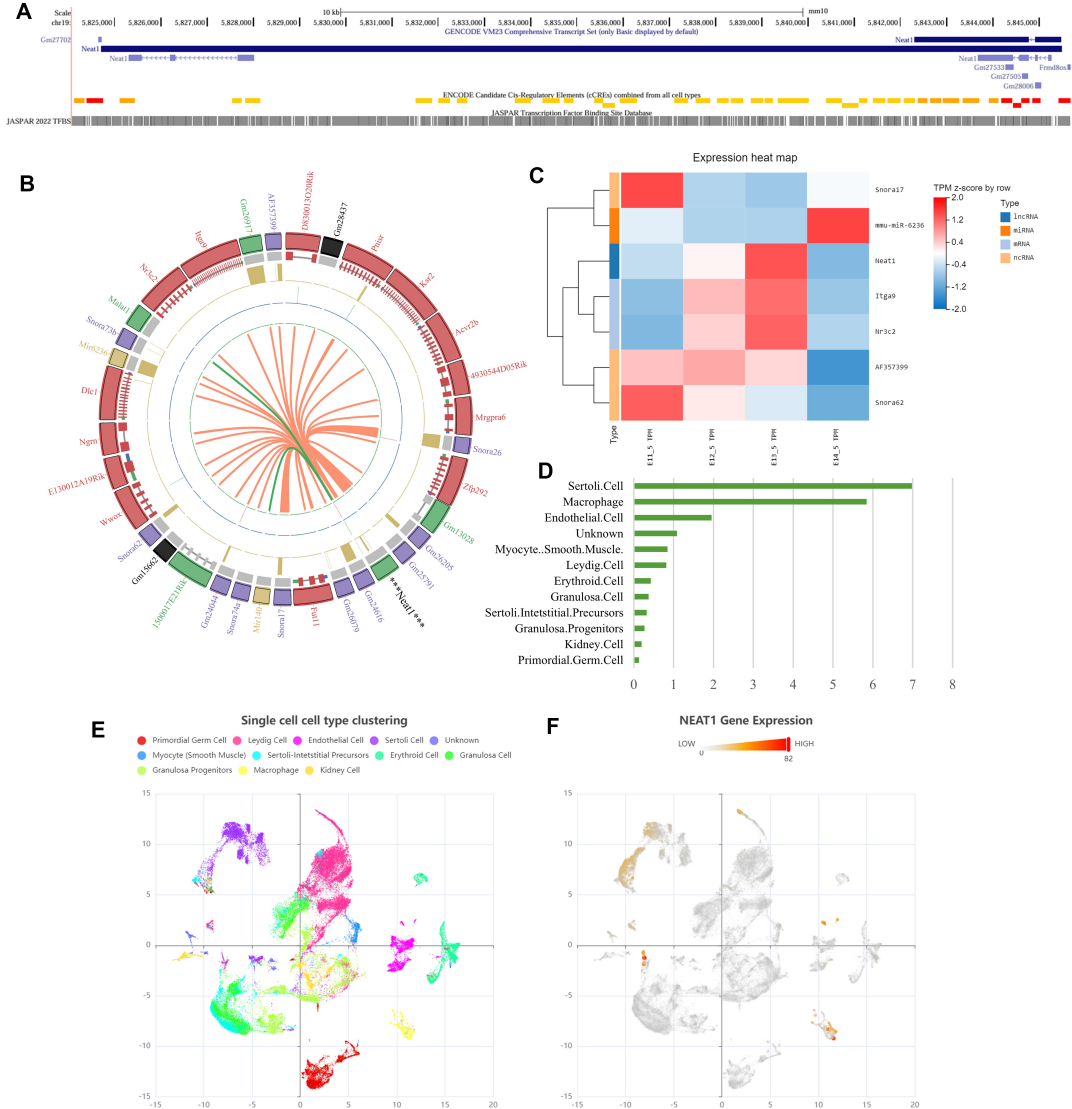
Characterization of DE lncRNAs. (**A**) The lincRNA *Neat1* localizes on Chr19 with multiple *cis-*regulatory elements and abundant TF-binding sites. (**B**) RNA–RNA interactome of *Neat1* identified from the RISE database. (**C**) Heatmap of *Neat1* and DEGs in its RNA–RNA interactome. *Neat1* showed a similar expression pattern to *Itga9* and *Nr3c2*, which was opposite to that of Snora17 and mmu-miR-6236. (**D–F**) *NEAT1* expression atlas in human fetal gonads from the TEDD database, which integrates scRNA-seq datasets, showed that *Neat1* was enriched in Sertoli cells.

During E13.5–E14.5, LOC102639045 was the most abundant and dramatically increased lncRNA that has been identified with the stefin gene cluster in the chromosomal ideogram (Fig. 2G). LOC102639350, 3930402G23Rik, and *Gm5600* were also up-regulated and showed a similar expression pattern during E11.5–E14.5. Most of the significantly down-regulated lncRNAs were expressed at a low level, including *Neat1*, 1110028F11Rik, LOC102639682, LOC102632032, and 4930563E18Rik. *Neat1* localizes on chromosome 19 and encodes nuclear paraspeckle assembly transcript 1 (Fig. 4A). It has been observed in samples of cervical, endometrial, and ovarian cancer [60]. Recently, interactions between *NEAT1* and DNA, miRNA, and proteins have been actively studied in carcinogenesis [61]. RNA–RNA interactions identified from the RISE database [62] showed that *Neat1* may have relationships with multiple RNAs (Fig. 4B), among which *Snora17*, mmu-miR-6236, *Itga9*, *Nr3c2*, *AF357399*, and *Snora62* were DEGs during E11.5–E14.5. *Neat1* showed a similar expression pattern to *Itga9* and *Nr3c2*, which was opposite to that of Snora17 and mmu-miR-6236, implying potential ceRNA networks (Fig. 4C). Predicted TF-binding sites on *Neat1* promoter and enhancer regions included *SRY*, *WT1*, *GATA4*, and *SOX9*. Data from published human scRNA-seq profiles showed that *NEAT1* was enriched in Sertoli cells [34, 63] (Fig. 4D–F), suggesting potential roles for *Neat1* in sexual development. However, the roles of *Neat1* and many other uncharacterized lncRNAs are still far from clear.

### Characterization of DE miRNAs

**Figure 5.**
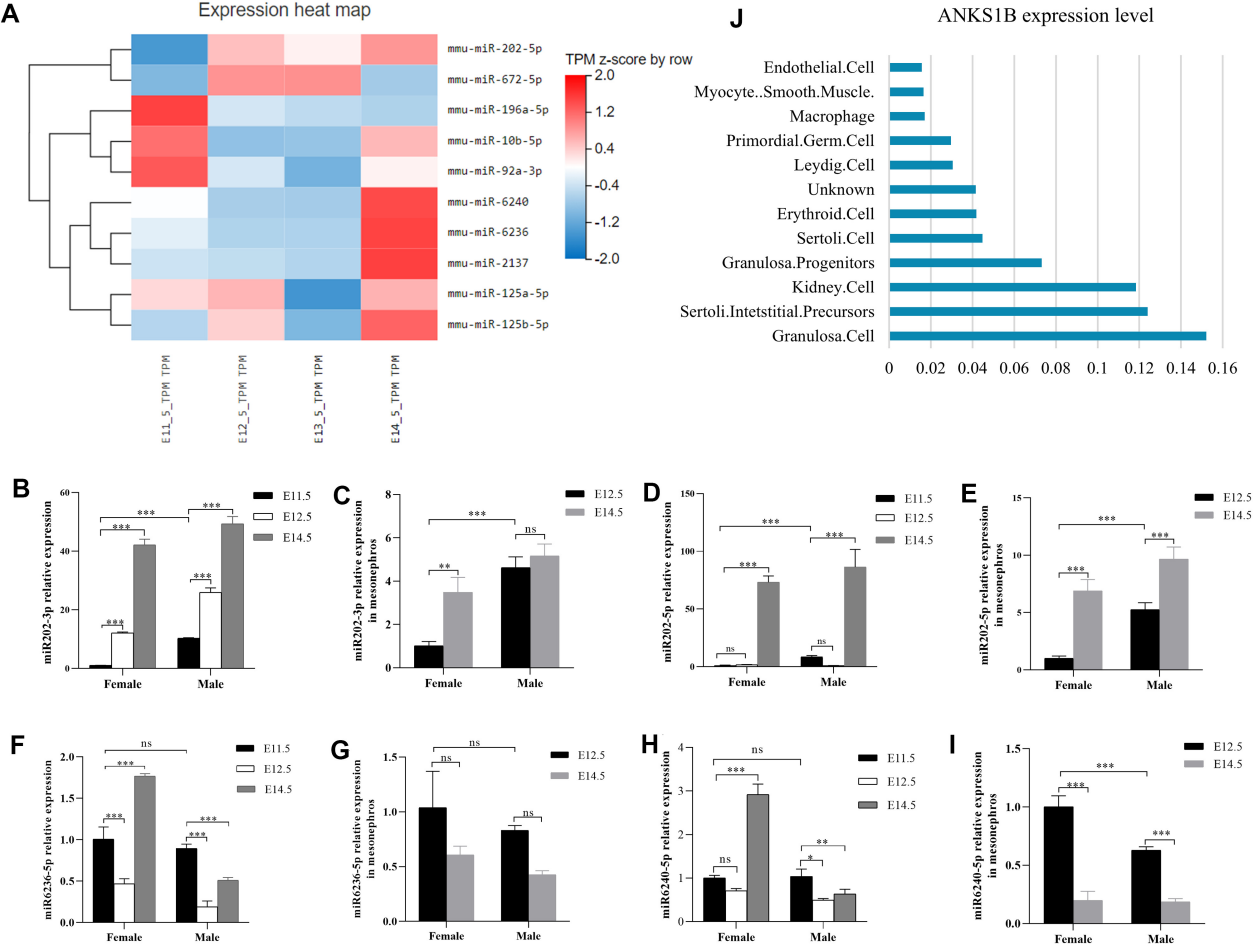
Characterization of DE miRNAs. (**A**) Expression heatmap of selected miRNA candidates. (**B–** **I**) Quantitative real-time PCR results of miR202-3p, miR202-5p, miR6236, and miR6240 in gonads and the mesonephros; *n* = 3. Statistical significance was determined using a two-way ANOVA. *P < 0.05, **P < 0.01, ***P< 0.001. (**J**) ANKS1B expression enrichment in human fetal gonads from the TEDD database.

Among the DE small ncRNAs, miR202 showed the greatest up-regulation, with a low expression level during early gonad development in our data. Elanor *et al.* have reported that the testis-determining factor gene *SOX9* regulates miR-202-5p/3p expression during mouse testis differentiation [64], and loss of miR-202 leads to apoptosis of spermatocytes [65]. Quantitative real-time PCR results validated that both miR-202-5p and miR-202-3p showed dramatic up-regulation in male and female gonads after E13.5, while their fold changes in the adjacent mesonephros tissue were smaller (Fig. 5B–E). miR-202-5p may contribute specifically to germ cell development while miR-202-3p may be involved in sex determination regulation as well. Another significantly up-regulated miRNA with logFC ≥ 2 from E11.5 to E12.5 was mmu-miR-672, which is located on the X chromosome. The most significantly down-regulated miRNAs included mmu-miR-6236, mmu-miR-196a-5p, and mmu-miR-6240. No DE miRNA was identified from E12.5 to E13.5. During E13.5–E14.5, all DE miRNAs showed up-regulation. The most significant ones included mmu-miR-2137, mmu-miR-6236, mmu-miR-6240, mmu-miR-125b, mmu-miR-125a, mmu-miR-3535, mmu-miR-92a, and mmu-miR-202-5p (Fig. 5A).

miR6236 and miR6240 were the most abundant DE miRNAs identified in our study and showed an interesting decrease from E11.5 to E12.5 followed by a significant increase from E13.5 to E14.5 in gonads (Fig. 5F–I). Down-regulation of miR6236 has been reported to enhance neuronal development and regeneration [66]. Our quantitative real-time PCR showed that the differential pattern of miR6236 expression was specific to the gonads but not the adjacent mesonephros (Fig. 5F–H). Both miR6236 and miR6240 showed higher expression in female gonads (Fig. 5F–I). Networks of miR6236 in the TransmiR v2.0 database revealed that miR6236 might interact with several TFs, including hormonal receptors *Ar*, *Esr1*, *Esrra* and *Esrrb*, *Sox* families, *Gata* families, *Dmrt1*, CTCF, *Ep300*, and several lysine demethylases (KDMs), much more complicated than networks of miR202 and miR6240. Conversely, target prediction based on TargetScan [29, 67], miRDB [68], and ENCORI databases [30] provides more candidate targets for miR6240 than for miR6236. Further detailed studies are needed to understand their specific networks and functions in cellular contexts.

Ankyrin repeat and SAM domain-containing protein 1B (*Anks1b*), a haploinsufficient gene predominantly expressed in the brain [69], was the only shared predicted target of miR6236 and miR6240 in the lists. The qPCR result confirmed that *Anks1b* was expressed at a low level and significantly up-regulated after E11.5 in female gonads (Fig. 7B, F). Analysis based on published scRNA-seq data in the TEDD database showed that expression of *Anks1b* was higher in female granulosa cells in humans (Fig. 5J). More interestingly, TF-binding sites of *Wt1*, *Gata*, and *Sox* families were found on promoter/enhancer regions of *Anks1b*, implying some interesting roles for miR6236/miR6240–*Anks1b*-related pathways downstream of gonad-specific TFs.

### Characterization of DE snoRNAs

Although the majority of stranded ncRNA biotypes maintained stable expression after sex determination, the number of DE snoRNAs increased significantly from 19 during E11.5–E12.5 to 86 during E13.5–E14.5, surpassing even the number of DE miRNAs. Out of the 86 DE snoRNAs, 31 were box H/ACA snoRNAs, which also included a small Cajal body-specific RNA (scaRNA) named Scarna3b, while the other 55 were box C/D snoRNAs, including Scarna3a.

**Figure 6.**
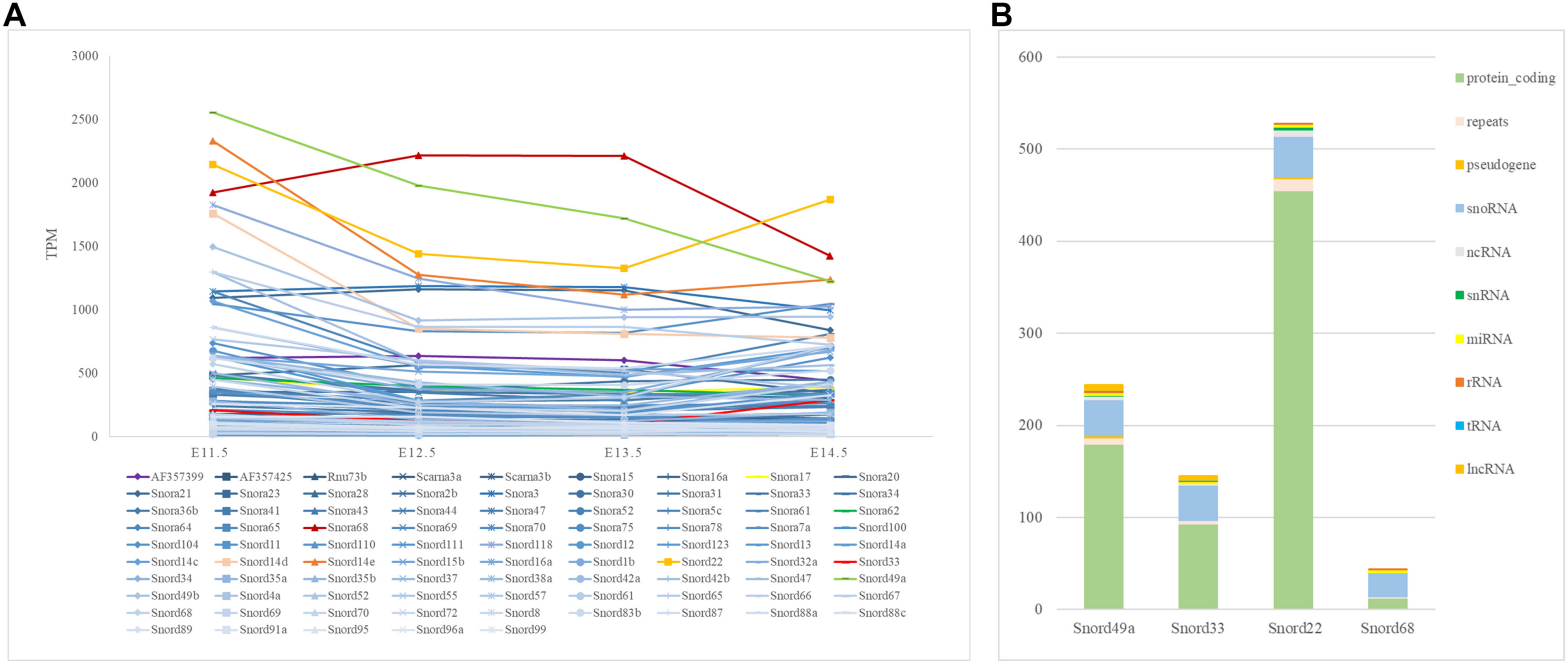
Characterization of DE snoRNAs. (**A**) Line chart showing the expression pattern of DE snoRNAs during E11.5–E12.5 based on TPMs. *Snord49a*, *Snord14e*, and *Snord22* were the most abundant snoRNAs, with >2000 TPMs from E11.5 to E12.5. During E13.5–E14.5, *Snord33*, expressed at a low level, showed the greatest up-regulation. *Snord22* and *Snora68* were the most abundant snoRNAs. (**B**) RNA–RNA interactions (RRIs) in the RISE database showed that *Snord22* has the most potential interactions with various RNA types. *Snord49a*, *Snord33*, and *Snord22* may mainly regulate mRNAs and then snoRNAs, while *Snord68* may interact mostly with other snoRNAs.

All the DE snoRNAs during E11.5–E12.5 showed ∼2-fold down-regulation, whereas the 86 snoRNAs in E13.5–E14.5 were 2- to 9-fold up-regulated. Among the DEG lists, *Snord49a*, *Snord14e*, and *Snord22* were the most abundant snoRNAs, with >2000 TPMs during E11.5–E12.5. No DE snoRNA was detected when comparing E13.5 with E12.5. During E13.5–E14.5, *Snord33*, expressed at a low level ,showed the greatest up-regulation. In addition, *Snord22* and *Snora68* were the most abundant snoRNAs. (Fig. 6A). We noticed that four DE snoRNAs, *Snord96a*, *Snord49b*, *Snord118*, and *Snord91a*, were located within the DE tRNA clusters on Chr11 (Fig. 2C). These snoRNAs may contribute more to the tRNA modifications.

RNA–RNA interactions (RRIs) in the RISE database showed that *Snord22* has the most potential interactions with various RNA types. *Snord49a*, *Snord33*, and *Snord22* may mainly regulate mRNAs and then snoRNAs, while *Snord68* may interact mostly with other snoRNAs (Fig. 6B). Interestingly, *Snord49a*, *Snord33*, *and Snord68* were all related to miR6240 based on the RISE database, suggesting some unknown interactions between the snoRNA clusters and miR6240.

### Identification of *Sox9* enhancer RNA with unassigned reads

Recent studies have revealed that active enhancers transcribe non-coding enhancer RNAs (eRNAs) that can interact with promoter–enhancer loops and contribute to the transcriptional regulation of surrounding protein-coding mRNAs [70–72]. Some eRNAs may not contain poly(A) tails [73]. In humans, duplication or deletion of core enhancers upstream of *SOX9* resulted in *SOX9* dose fluctuations and sex reversal [74]. In mouse sex determination, deletion of the testis-specific enhancer core sequence (TESCO) reduced ∼50% of *Sox9* expression in XY fetal gonads [75].

**Figure 7.**
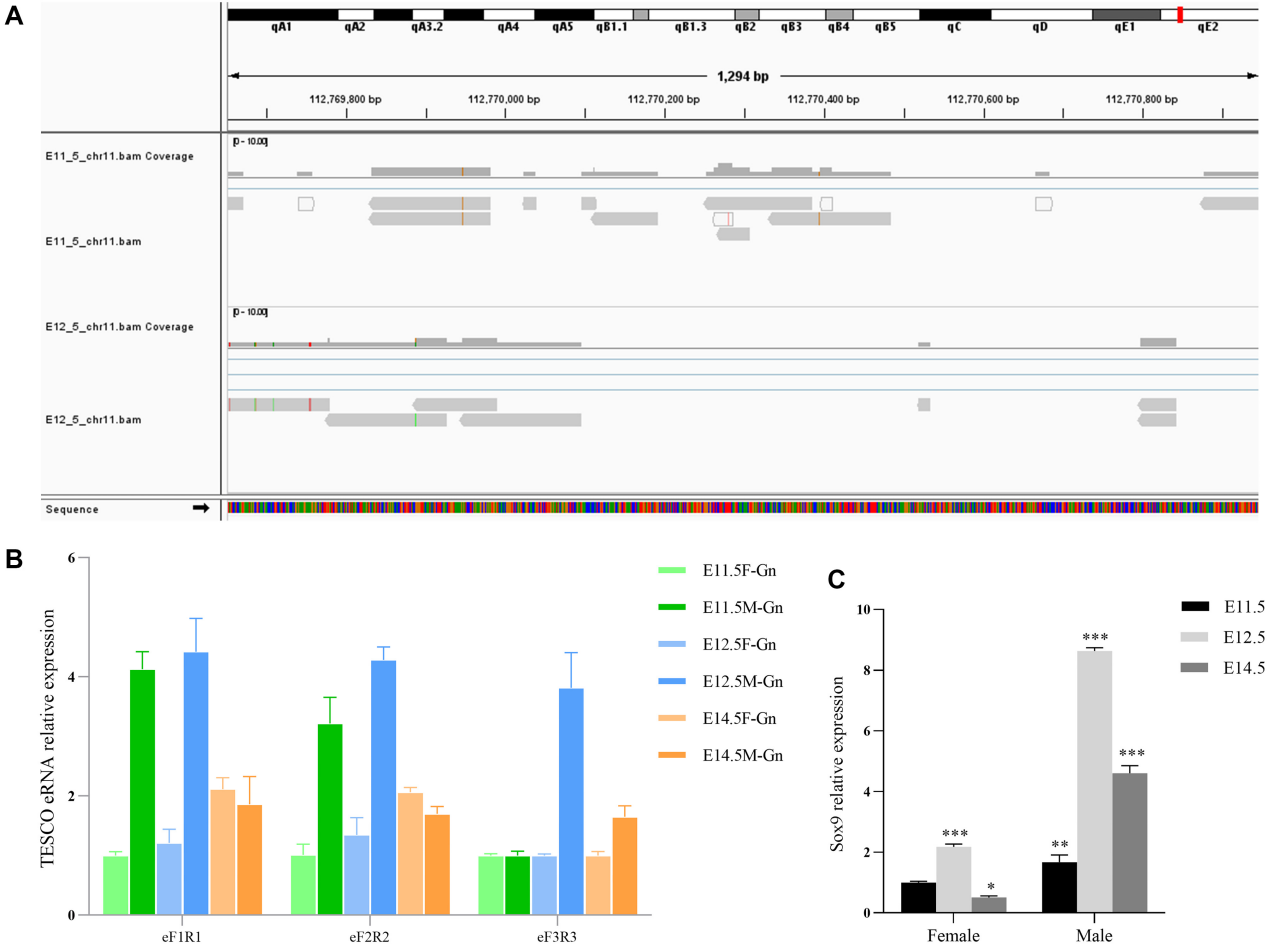
Identification of Sox9 enhancer RNA with unassigned reads. (**A**) Visualization of unassigned reads mapped to the TESCO enhancer region (Chr11: 112 769 652–112 770 944, mm10) with IGV. (**B**) Quantitative real-time PCR results from three primer pairs confirmed the expression of TESCO eRNA, which showed the highest expression in E12.5 male gonads. (**C**) *Sox9* also showed the highest expression in E12.5 male gonads from the qPCR result.

Attempting to extend the advantage of our data, which cover complete stranded RNAs with or without a poly(A) tail, we visualized the bam files of the 1293 bp TESCO region (Chr11: 112 769 652–112 770 944, mm10) with IGV software and found a few unassigned reads mapped to the enhancer (Fig. 7A). Quantitative real-time PCR results from three pairs of primers confirmed the higher expression of TESCO eRNA at E12.5 (Fig. 7B), which is consistent with the *Sox9* transcription pattern in male gonads (Fig. 7C). Of note, two lncRNAs, TCONS_00 025 195 and TCONS_00 025 196, have been identified from the *RevSex* region (517–595 kb 5′ to *SOX9*) that contained important distal enhancers of *SOX9* in human testes [76], suggesting that lncRNAs transcribed from enhancer regions may play certain roles in *SOX9* regulation. About half of the long unique mapped reads were unassigned to genes on the mm10 reference genome, suggesting a potential large amount of unidentified non-coding stranded RNAs in development.

### Potential regulatory networks in early gonad development

**Figure 8.**
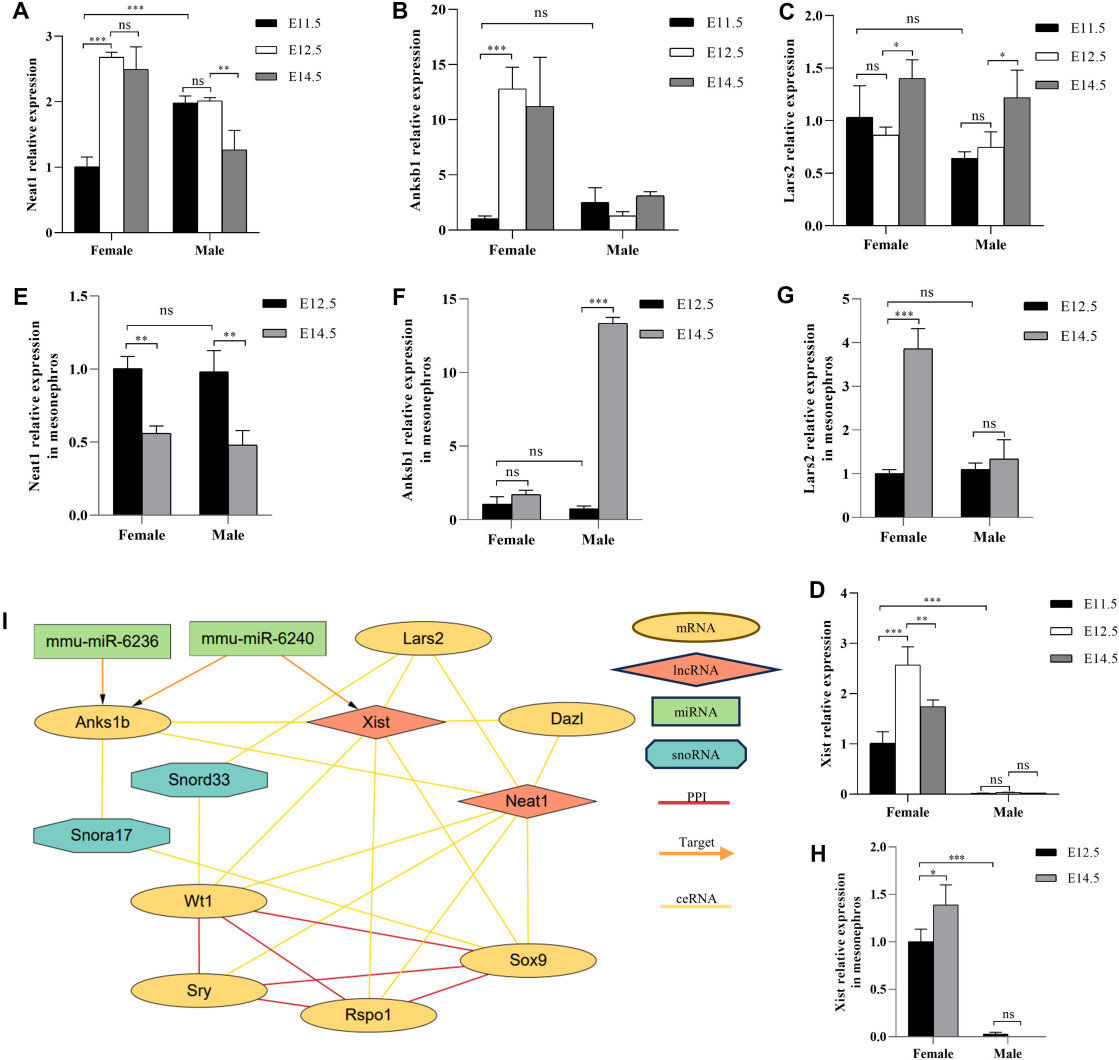
mRNA–lncRNA–small RNA network construction. (**A**) Quantitative real-time PCR of candidate genes *Anks1b*, *Neat1*, *Lars2*, and *Xist* in male and female gonads and mesonephros; *n* = 3. Error bars are the SD. *P*-values derived from two-way ANOVA: *P < 0.05, **P < 0.01, ***P < 0.001; ns indicates not significant. (**B**) Visualization of RNA networks with Cytoscape. PPI prediction was based on STRING11. Target relationships between miRNA and mRNA/lncRNA were supported by at least two tools from TargetScan, miRDB, RNAhybrid, and ENCORI. Predicted ceRNA relationships were calculated with the Dr. Tom system based on the ratio of shared miRNAs of selected mRNA/lncRNA.

To gain a better understanding of the dynamic RNA regulatory networks, miR6236, miR6240, *Snora17*, *Snord33*, *Neat1*, *Anks1b*, *Xist*, *Lars2*, *Wt1*, *Sry*, *Sox9*, *Rspo1*, and *Dazl* were selected for target, PPI, and ceRNA network analysis. qPCR relative expression patterns showed that *Xist*, *Neat1*, and *Anks1b* had similar up-regulation and down-regulation in female gonads, which is the opposite to the patterns of miR6236 and miR6240. *Lars* demonstrated significant up-regulation after E12.5 in both male and female gonads (Fig. 8A–D). However, the expression patterns in the mesonephros varied greatly. *Xist* and *Lars2* showed differential expression in the female mesonephros, whereas *Anks1b* was significantly up-regulated in the male mesonephros at E14.5. Furthermore, *Neat1* displayed an identical pattern in the male and female mesonephros (Fig. 8E–H).

miR6236 and miR6240 were both predicted to target *Anks1b*. *Xist* was also a candidate target of miR6240 and showed ceRNA linkages with *Anks1b* and *Lars2*, suggesting the potentially important roles of these genes in female gonad differentiation, especially in granulosa cells. *Anks1b* also showed ceRNA linkage with *Neat1*, while *Neat1* and *Xist* showed active ceRNA networks with the TFs (Fig. 8I).

*Snora17* showed ceRNA relationships with *Sox9* and *Anks1b*, implying that Snora17 might also be involved in supporting lineage differentiation. In contrast, *Snord33* showed ceRNA networks with *Wt1* and *Lars2*, which are commonly expressed in gonadal cells. The functional networks of snoRNAs are still difficult to characterize well. A better understanding of their secondary structures is necessary to identify direct interactions.

## Discussion

Aiming to profile more comprehensive transcriptomic dynamics during early gonad development, including sex determination and germ cell development stages, we collected wild-type mouse gonads at E11.5–E14.5 and performed complete stranded RNA-seq. By adding poly(A) tails as well as UMI to all stranded RNAs followed by RNA enrichment with oligo(dT)s during sequencing library construction, we were able to explore the unbiased expression data of long and short stranded RNAs with or without poly(A) tails in one preparation. Previous transcriptome profiling focusing on mRNA and lncRNA with poly(A) tails required a separate small RNA library for short RNA expression data [8, 77, 78]. Thus, RNAs without poly(A) tails were less annotated and studied. What is more, the construction of separate libraries may lead to bias when analyzing relationships between the long and short RNAs. Constructing two kinds of NGS libraries is also less cost-effective.

Our data recovered 30 462 long RNAs and 635 miRNAs from about half of the unique mapped reads. The other half of the reads were unassigned to known genes in the mm10 reference genome, suggesting a potentially large amount of uncharacterized ncRNAs in mammals. Unassigned sequencing reads are not uncommon in NGS data. Some RNA reads may be derived from technical artifacts such as sequencing errors [79]. The limitation of the mm10 reference genome is also an explanation since the genome projects were primarily focused on the protein-coding region that makes up only 1–2% of mammalian genomes [80]. To address this limitation, the development of a more comprehensive reference genome for non-coding regions will be crucial. Furthermore, comparison with datasets obtained from different profiling methods will be helpful to improve the accuracy of the analysis results.

In our study, 2419 DEGs were identified from E12.5 versus E11.5, and 333 and 770 DEGs were identified from E13.5 versus E12.5 and E14.5 versus E13.5, respectively. The DEG lists only have 37 overlapping genes. Some of the genes showed up-regulation and then down-regulation patterns during E11.5–E14.5, suggesting highly sophisticated temporal regulation during sex determination and germ cell development. The interesting fluctuations in expression may be associated with processes such as cell proliferation, migration, and the establishment of specialized cell lineages in gonads. Most of the DEGs after E13.5 were up-regulated, demonstrating that germ cell development relies on a new wave of transcriptome activation, which is consistent with previous reports [81, 82].

Ideograms of the distribution of DEGs on each chromosome showed that Chr6 and Chr2 might be more essential in early gonadogenesis and sex determination, while Chr11 and Chr13 might be the most active chromosomes during mouse early gonad development, especially for germ cell development. These active chromosomes may serve as key targets for epigenetic modifications and advanced chromatin conformation research during early gonad development. In evolutionary biology, these results can also help to compare the dynamic differences and similarities at the chromosome level in the sex determination process between species. We further identified active regional divisions on the chromosomes, including Hox and stefin genes. Some ncRNAs are embedded within these coding gene clusters. However, how the proximal ncRNAs contribute to the regional chromatin remodeling and coding gene expression remains mostly unclear [83, 84]. The tRNA clusters also showed significant regional enrichment, including some snoRNAs interspersed among them. snoRNA–tRNA interaction might be required for global tRNA modifications and codon usage [85], further affecting protein production. Our findings open up more possibilities in RNA research, developmental, and reproductive biology, and will advance prenatal screening and diagnosis, and genetic counseling which currently rarely incorporate non-coding regions [38].

The DEGs can be classified into 22 RNA types. tRNAs, lncRNAs, antisense RNAs, snoRNAs, and miRNAs were the major DE RNAs in addition to protein-coding mRNAs. We identified coding genes that have been related to reproductive disorders but not well studied in development, including *Lars2* and *Gykl1* that are related to mitochondria. scRNA-seq data suggested that *Lars2* was enriched in granulosa cells in fetal gonads [34], implying that it may play an essential role in ovary differentiation and maturation. Additionally, our data showed a transition of the main globin in gonads, which was consistent with previous knowledge of globin switches in liver, heart, and bone marrow research [86, 87]. Levels of globin and angiogenesis might vary in male and female gonad development [88]. Globins may also have non-oxygen-related functions, such as antioxidant properties or involvement in signaling pathways [89].

tRNA was the second most differentially expressed RNA class. The 158 DE tRNAs can be categorized into 16 groups according to the amino acids they carry. Twenty-three DE tRNAs were responsible for arginine, comprising the largest portion of the DE tRNAs (Table 2). n-Te15, n-Te18, n-Te21, and Trnae-uuc that transport lysine were the most abundant tRNAs. Modifications of lysine and arginine residues on histone could lead to chromatin remodeling during germ cell development [90, 91]. Dysregulation of tRNAs has been related to genetic disorders and cancer. Pioneering and innovative therapeutic strategies targeting tRNA are increasingly gaining attention [92]. They may also be potential biomarkers and therapeutic targets in reproductive health.

While studies on lncRNA in hermaphroditic species have demonstrated some roles for lncRNAs in sex determination [93], the specific mechanisms of pseudogenes, lncRNA, and antisense RNA in human and mouse gonad development have not been extensively studied. Pseudogenes were generally believed to be non-functional. We found thart the DEG *Gm15529*, HORMA domain-containing 1 pseudogene, was predominantly detected in mouse gonads at and after E11.5, implying its potential role specifically in gonad development. DE antisense RNAs such as 4833422C13Rik and 0610038B21Rik were barely documented. Antisense transcripts of TF genes such as *SOX9* and *WT1* have been shown to have functional roles in cancer [94, 95]. They can regulate gene expression through transcriptional interference, RNA masking, or RNA stability modulation [96]. The DE antisense transcripts may also be active in mammalian gonad development through various mechanisms. The lncRNA LOC102635594 (Gm32885) was significantly up-regulated from E11.5 to E12.5, and it showed biased expression specifically in the placenta and testis of adult mice in the Mouse ENCODE project [54], suggesting that it may function in maternal and embryonic signaling transduction and testis differentiation. *Neat1* showed significant down-regulation after E13.5. Several characteristics of *Neat1* led to the hypothesis that *Neat1* together with snoRNA and miRNA may contribute to Sertoli cell maturation in the male gonad. The lncRNAs may act as molecular scaffolds, interacting with chromatin and TFs to modulate gene expression and influence cell fate decisions [97]. Various uncharacterized DE lincRNAs were also found on sex chromosomes, further demonstrating the complexity of the RNA world during sexual development.

Transcriptomic profiling based on different methods has identified more and more small RNAs in reproductive tracts [98–101]. miRNAs are the most widely studied small RNA types. Consistent with earlier studies, we found that miRNA202 [64], miR10 [102], miR125 [103], miR196 [104], and the let-7 family [105] were differentially expressed during mammalian early gonad development. We also identified two new DE miRNAs, miR6236 and miR6240, both of which were abundant but not well characterized in the gonad yet. miR6236 has been shown to be involved in neuronal development [66], heart development [106], skeletal muscle angiogenesis [107], and cancer progression [108] in recent years. Our results confirmed the down-regulation and then up-regulation pattern of miR6236 to be specific to the gonads but not to the adjacent mesonephros during E11.5–E14.5. miR6240 has been linked to Alzheimer's disease [109] and cardiomyocyte proliferation and regeneration [110]. It showed a pattern of down-regulation and subsequential up-regulation in both gonads and mesonephros, suggesting that it may play a more complex role in development of the complete reproductive tract. *Anks1b* was the predicted target of both miR6236 and miR6240, which showed enrichment in female granulosa cells from human fetal gonad scRNA-seq, further suggesting the involvement of the miRNA6236 network in supporting gonadal cell development. TF-binding sites of *Wt1*, *Gata*, and *Sox* families were found on promoter/enhancer regions of *Anks1b*, implying that miR6236/miR6240–*Anks1b* networks might be downstream of gonad-specific TFs.

Earlier research on snoRNAs has shed light on their functional significance, including their involvement in controlling codon-biased dichotomous cellular states and global tRNA modifications [85]. Structural features are important aspects related to RNA functions. The different numbers of dynamic box C/D and box H/ACA snoRNAs implied that the snoRNAs may be mainly involved in rRNA 2′-*O*-ribose methylation during gonad development. In recent years, the roles of snoRNAs have expanded beyond tRNA and rRNA modifications. They could also be involved in alternative splicing, as well as snoRNA-derived small fragments with miRNA-like functions [18]. However, research on the molecular mechanism of most snoRNAs is very limited.

Significant bias exists in different library construction methods even with identical samples [78]. Unexpectedly, PIWI-interacting RNAs (piRNAs) which are known to suppress transposable elements (TEs) in the germline [111] were not detected in our sequencing data. Diversification of piRNA expression during early gonad development has been reported in recent years [112, 113]. The failure to detect piRNAs may be due to technical differences in the library construction methods. Samples were fractionated by electrophoresis in acrylamide gels or oxidized by NaIO_4_ for piRNA enrichment during library construction in some previous studies [114–116]. The enzyme we used may not be sufficient to ligate poly(A) tails onto piRNAs due to unknown modifications or secondary structures [117]. Thus, the piRNAs were lost in the subsequent enrichment of polyadenylated RNAs with oligo(dT)s. The limitation of bioinformatic algorithms and the piRNA database may also be a reason [118]. Data obtained by different methods can complement each other to a certain extent.

In RNA network construction, we selected miR6236, miR6240, *Snora17*, *Snord33*, *Neat1*, *Anks1b*, *Xist*, *Lars2*, *Wt1*, *Sry*, *Sox9*, *Rspo1*, and *Dazl* for target, PPI, and ceRNA network analysis. The results suggested that miR6236/miR6240–*Anksb1*–*Neat1* might be involved in gonadal supporting lineage differentiation. *Anks1b* might interact with *Xist* in female granulosa cells, while *Neat1* functions in male Sertoli cells. Both *Anks1b* and *Neat1* might be downstream of TFs known to be critical in sex determination and somatic cell differentiation. *Neat1* showed ceRNA linkage with the leucyl-tRNA synthetase-encoding *Lars2* which has been related to gonadal dysgenesis. *Lars2* and *Wt1* shared ceRNA linkages with *Snord33*. *Anks1b* and *Sox9* shared ceRNA relationships with *Snora17*. These implied the presence of complex RNA networks including tRNAs, snoRNAs, lncRNAs, and TFs in gonad development. The various patterns of these genes in the mesonephros suggested that their involvement in reproductive tract development goes beyond the gonads.

Since RNA was first described as a key player in the “Central Dogma of Molecular Biology” by Crick in the mid-20th century [119], research on discovering RNA characteristics and function has been actively emerging, much of which has reshaped our knowledge of the genetic and epigenetic world. Successful RNA vaccines and RNA therapy development have demonstrated the robustness and unlimited prospects of RNA [120, 121]. In this study, we reported the first comprehensive profiling of stranded RNAs encompassing mainly lncRNAs, miRNAs, snoRNAs, tRNAs, and mRNAs during early mouse gonad development. The long list of uncharacterized DE RNAs suggested a huge gap between our current knowledge of the RNA world and the actual complexity of RNA molecules in the developing gonad, and will possibility open up new avenues in the future for prenatal screening and diagnosis, genetic counseling, and therapeutic interventions for reproductive disorders.

## Supplementary Material

ugaf014_Supplemental_Files

## Data Availability

The RNA-seq data in this study have been deposited in the NCBI Gene Expression Omnibus (GEO) under accession number GSE256154.
