# Supplementary material for: Complete stranded RNA profiling during early mouse gonad development
Source: NAR Mol Med. 2025 May 2;2(2):ugaf014. doi: 10.1093/narmme/ugaf014 (PMC12430024; doi:10.1093/narmme/ugaf014)
Supplement: ugaf014_Supplemental_Files [file ugaf014_Supplemental_Files.zip › Supplementary Table 4. Numbers of DEGs distributed on each chromosome.pdf]

Supplementary Table 4. Numbers of DEGs distributed on each chromosome

	Counts of DEGs		
	E11.5-E12.5	E12.5-E13.5	E13.5-E14.5
chr1	168	25	51
chr2	199	29	50
chr3	139	19	41
chr4	147	18	41
chr5	137	20	33
chr6	135	15	27
chr7	177	24	53
chr8	123	24	39
chr9	126	16	37
chr10	106	12	24
chr11	179	26	83
chr12	68	13	22
chr13	114	20	67
chr14	90	8	30
chr15	109	11	35
chr16	63	9	23
chr17	79	17	32
chr18	63	7	16
chr19	69	7	16
chrX	124	13	41
chrY	4	0	2
chrMT	0	0	6
Unknow	0	0	1
**Grand Total**	**2419**	**333**	**770**
