# Supplementary material for: Complete stranded RNA profiling during early mouse gonad development
Source: NAR Mol Med. 2025 May 2;2(2):ugaf014. doi: 10.1093/narmme/ugaf014 (PMC12430024; doi:10.1093/narmme/ugaf014)
Supplement: ugaf014_Supplemental_Files [file ugaf014_Supplemental_Files.zip › Supplementary Table 5. List of PCR primers.pdf]

Supplementary Table 5. List of PCR primers

Primer name	Primer sequence
miRNA-RT-primer	CAGGTCCAGTTTTTTTTTTTTTTTVN
mmu-miR202-3p-F	GAGAGGTATAGCGCATGG
mmu-miR202-3p-R	CCAGTTTTTTTTTTTTTTTCTTCCCA
mmu-miR202-5p-F	CGCAGTTCCTATGCATATACTTC
mmu-miR202-5p-R	AGGTCCAGTTTTTTTTTTTTTTTAAAGA
mmu-miR6236-5p-F	GTCGCCGGCAGTC
mmu-miR6236-5p-R	GGTCCAGTTTTTTTTTTTTTTTCCT
mmu-miR6240-5p-F	CCAAAGCATCGCGAAGG
mmu-miR6240-5p-R	GTTTTTTTTTTTTTTTCGCCGTG
U6-F1	GGAACGATACAGAGAAGATTAGC
U6-R1	TGGAACGCTTCACGAATTTGCG
Sox9-F	AGGAAGCTGGCAGACCAGTA
Sox9-R	CGTTCTTCACCGACTTCCTC
eTESCO-F1	TCGGCCTTTGTTCCTAACCT
eTESCO-R1	CACTTGTGTTTGACCCTGGG
eTESCO-F2	GGCTGGCCTTTCTCTCTCTT
eTESCO-R2	ACTTGATGCTCTTGGGGTGA
eTESCO-F3	GTATCCTTGTCCCACCTCCC
eTESCO-R3	GCTGGTGGAGATTGCAGAAC
Neat1-F	CAGGAGGCCATCGTTGAAGT
Neat1-R	CCATGAAAAAGGGTGCCTGC
Xist-F	AATGGAACGGGCTGAGTTTTAG
Xist-R	TCATCCGCTTGCGTTCATAG
Anks1b-F	CCACCTCTTCTGTGGAAACCAC
Anks1b-R	CCTGCGAATTGCCCAGCATCAT
Lars2-F	CATAGAGAGGAATTTGCACCCTG
Lars2-R	GCCAGTCCTGCTTCATAGAGTTT
mGapdh-F	AGGTCGGTGTGAACGGATTTG
mGapdh-R	TGTAGACCATGTAGTTGAGGTCA

V is A, C, and G and N is A, C, G, and T (Sigma).
